# Cryogel-Templated Fabrication of n-Al/PVDF Superhydrophobic Energetic Films with Exceptional Underwater Ignition Performance

**DOI:** 10.3390/molecules27206911

**Published:** 2022-10-14

**Authors:** Jingwei Li, Xuwen Liu, Quanmin Xie, Yongsheng Jia, Jinshan Sun, Yingkang Yao

**Affiliations:** 1 State Key Laboratory of Precision Blasting, Jianghan University, Wuhan 430056, China; 2 Hubei Key Laboratory of Blasting Engineering of Jianghan University, Wuhan 430056, China; 3 School of Chemistry and Chemical Engineering, Nanjing University of Science and Technology, Nanjing 210094, China

**Keywords:** nanoenergetic materials, superhydrophobic surface, anti-aging, underwater ignition, thermal properties, polymeric composites

## Abstract

The rapid heat loss and corrosion of nano-aluminum limits the energy performance of metastable intermolecular composites (MICs) in aquatic conditions. In this work, superhydrophobic n-Al/PVDF films were fabricated by the cryogel-templated method. The underwater ignition performance of the energetic films was investigated. The preparation process of energetic materials is relatively simple, and avoids excessively high temperatures, ensuring the safety of the entire experimental process. The surface of the n-Al/PVDF energetic film exhibits super-hydrophobicity. Because the aluminum nanoparticles are uniformly encased in the hydrophobic energetic binder, the film is more waterproof and anti-aging. Laser-induced underwater ignition experiments show that the superhydrophobic modification can effectively induce the ignition of energetic films underwater. The results suggest that the cryogel-templated method provides a feasible route for underwater applications of energetic materials, especially nanoenergetics-on-a-chip in underwater micro-scale energy-demanding systems.

## 1. Introduction

Metastable intermolecular composites (MICs) usually consist of nano-sized active fuels (e.g., Al) and oxidants as a system [1,2,3]. It has many applications in the water environment, including underwater propulsion, blasting, and welding [4,5,6]. However, the underwater application of MICs is mainly limited by the reaction environment [7,8]. It is not merely that water disturbs the reactants before ignition. Additionally, once ignited, the heat quickly dissipates into the surrounding water, making the combustion reaction difficult to maintain. More significantly, nanoscale aluminum readily reacts with water, significantly degrading the performance of MICs [9,10]. As a result, conducting in-depth study on the fabrication and characteristics of superhydrophobic MICs is critical.

In recent years, many efforts have been devoted to the field of superhydrophobic MICs [9,11]. A common parameter used to quantify surface hydrophobicity is the contact angle [12]. There are several contact angle models that can explain the liquid–solid interface state. Wenzel [13] believes that part of the liquid penetrates completely into the microstructure on the solid substrate, and the surface roughness amplifies the hydrophobicity of the hydrophobic surface. Cassie [14] believes that droplets on hydrophobic surfaces do not fill the grooves because air remains at the bottom of the grooves. Liu considers wetting phenomena on general substrates from a new perspective of continuum mechanics and relates film roughness to film contact angles based on triple contact lines (TCL), which has obtained numerous applications [15]. Regardless, superhydrophobic surfaces require a combination of desired roughness and low surface energy [16,17]. Therefore, in MICs systems, low surface energy is often provided by fluoropolymers (e.g., PVDF, FAS) [18,19]. At the same time, thanks to the strong electronegative fluorine–metal reaction (such as the Al–F reaction) [20,21], these fluoropolymers have low surface energy and strong oxidizing properties, which can help the energy release of the MICs system [22,23].

For the micro- and nano-scale roughness, there are also many methods, such as magnetron sputtering and vapor deposition for realizing the special microstructure of MICs [11]. However, these studies have their limitations. On the one hand, the MICs composites added with fluoropolymer coating rapidly lose heat to the aqueous environment before the self-propagation of the reaction, which restricts the release of energy. On the other hand, high production cost and low efficiency also limit its wide application.

Polyvinylidene fluoride (PVDF) has successfully attracted widespread attention as a material with good processability, low surface energy, low cost, and high fluorine content. In this work, we took advantage of the low surface energy and strong oxidizing properties of PVDF. A method for preparing n-Al/PVDF energetic thin films based on cryogel-template method is proposed. The morphology, water contact angle, anti-aging ability, thermal behavior, and underwater laser ignition performance of the energetic film were investigated.

## 2. Results and Discussion

### 2.1. Morphology

**Figure 1:**
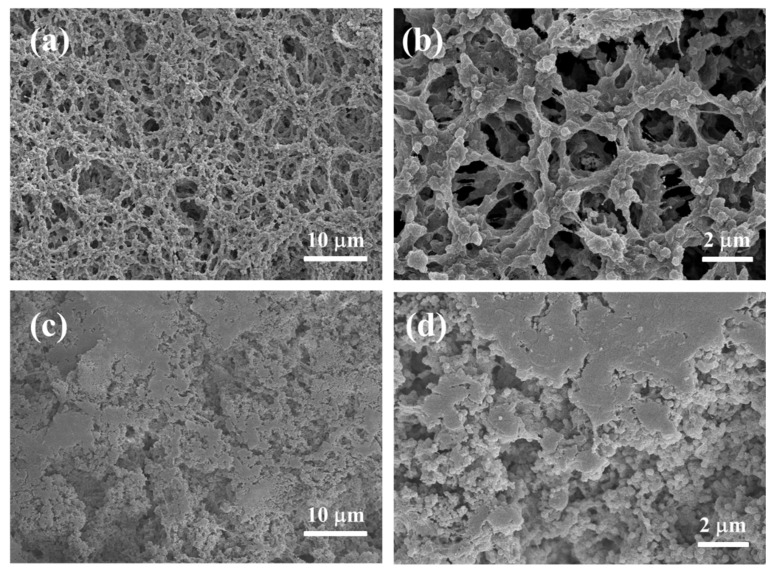
Top-view SEM images of I-Al/PVDF (**a**,**b**) and M-Al/PVDF (**c**,**d**).

The top-view SEM images of I-Al/PVDF and M-Al/PVDF are shown in Figure 1. The PVDF in I-Al/PVDF is nanofibrous and crosslinked with each other in the form of nano-network. The Al NPs are coated with PVDF and embedded in the PVDF nano-network, as shown in Figure 1a,b. The main reason for the formation of this morphology is that the disordered PVDF molecular chains in the homogeneous polymer solution first form a block during the phase transition in the coagulation bath, forming a porous polymer gel [24,25]. In PVDF gel, as ice crystals form, the solidification front collides with PVDF gel to form macroscopic grooves between growing ice crystals and particles trapped between ice branches, resulting in the formation of a 3D network structure during freezing [26,27].

**Figure 2:**
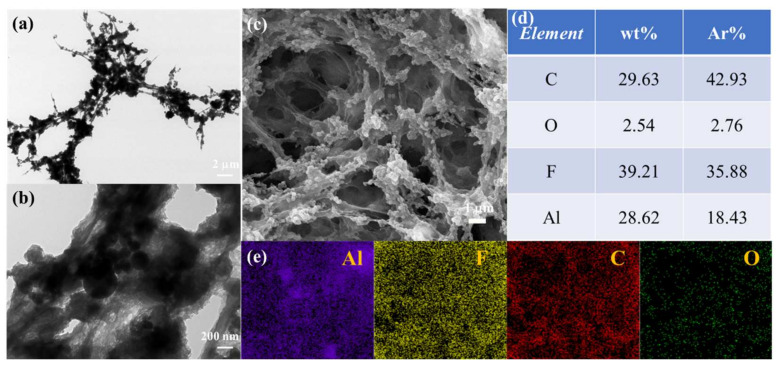
(**a**,**b**) TEM analysis of I-Al/PVDF fragmentation; (**c**) The original SEM image of EDS area of I-Al/PVDF; (**d**,**e**) Elemental mapping images of Al, F, C, and O species.

TEM characterization corroborates the information provided by SEM characterization, as shown in Figure 2a,b, where n-Al is encapsulated by PVDF. Al, C, O, and F element distributions are shown in Figure 2c–e. Elemental distribution of the n-Al/PVDF film, indicating that the components are well dispersed. In the samples without cryogelation (M-Al/PVDF), as shown in Figure 2c,d, the n-Al agglomerates seriously. The n-Al is mostly bare and does not combine well with PVDF. Whereas PVDF exists in a dense bulk form with low porosity in the film. This indicates that cryogel-fabricated n-Al/PVDF films have more regular morphology, in which Al NPs are more tightly bound to PVDF.

### 2.2. Water Contact Angle

**Figure 3:**
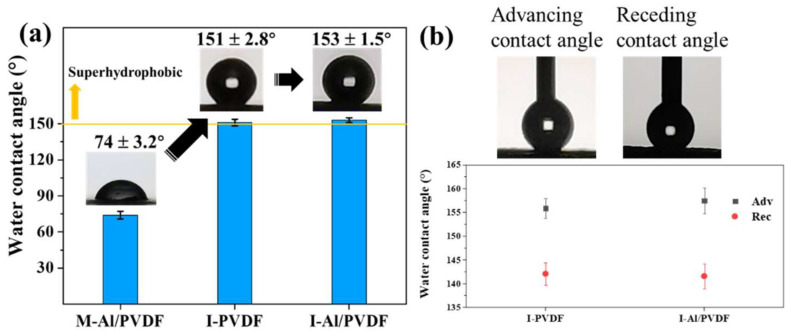
(**a**) The static water contact angle and (**b**) advancing/receding angles of each sample.

The static water contact angle results are shown in Figure 3a. Al NPs are generally considered to be hydrophilic due to the presence of hydroxyl groups in Al_2_O_3_ on the Al surface. However, with the addition of PVDF, the surface of M-Al/PVDF increases the hydrophobicity. The contact angle of the I-PVDF and I-Al/PVDF is 151 ± 2.8° and 153 ± 1.5°, realizing super-hydrophobicity. This is consistent with the results that the PVDF gel block is microscopically squeezed during the process of water crystallization, resulting in microscopic roughness [26,27]. The advancing/receding contact angles of the samples prepared from cryogel are shown in Figure 3b, the advancing/receding contact angles of I-PVDF and I-Al/PVDF are 156 ± 2.1°/142 ± 2.7° and 158 ± 2.4°/140 ± 2.6°, respectively. The contact angle hysteresis is 14° and 18°, respectively. This indicates the non-stickiness of the sample surface. In the meantime, this also illustrates that the cryogel method is an effective method to impart superhydrophobic surfaces to PVDF-based energetic materials.

### 2.3. Anti-Aging

**Figure 4:**
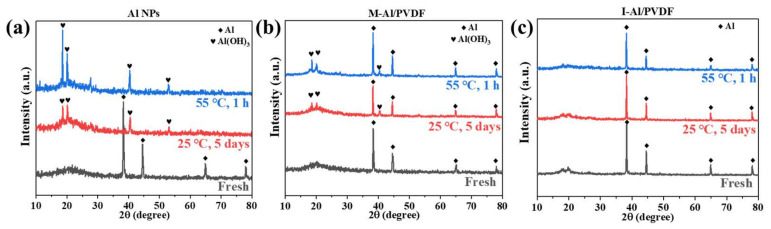
XRD patterns of Al NPs (**a**), I-Al/PVDF (**b**) and M-Al/PVDF (**c**) in the aging experiment.

Aging resistance is a crucial characteristic of energetic materials because it shows how well their energy density is maintained. As for the corrosion resistance for water, two groups of contrastive experiments have been conducted: one is that the Al NPs or Al/PVDF films are put into deionized water for 5 days at room temperature (25 °C), another is that the Al NPs or Al/PVDF films are put into deionized water for 1 h at high temperature (50 °C), as shown in Figure S2. Each sample’s XRD patterns after aging and vacuum drying are displayed in Figure 4. For Al NPs, corresponding diffraction peaks were observed at 38.5°, 44.7°, 65.1°, and 78.2° (JCPDS 04-0787) under both room temperature and high temperature aging conditions, as shown in Figure 4a. The distinctive diffraction peaks of Al vanish. Although this is happening, the characteristic Al(OH)_3_ diffraction peaks arise at 18.8°, 20.4°, 40.6°, and 53.1° (JCPDS 20-0011). This implies that aluminum has a propensity to react with water in an aquatic environment and lose its “fuel”-like qualities.

Although the diffraction peaks of Al (38.5°, 44.7°, 65.1°, and 78.2°) persisted after M-Al/PVDF was stored in water under both circumstances, portion of the diffraction peaks of Al(OH)_3_ (18.8°, 20.4°, 40.6°, and 53.1°) were seen, as shown in Figure 4b. This implies that, in comparison to Al NPs, the addition of hydrophobic PVDF can lessen the contact of aluminum with water in the environment, making it resistant to aging. However, a little portion of Al that is not protected by PVDF combines with water to create Al(OH)_3_.

The diffraction peaks of Al (38.5°, 44.7°, 65.1°, and 78.2°) for I-Al/PVDF are still present after storage in an aqueous environment under both conditions. However, no peaks of Al(OH)_3_ were seen, as shown in Figure 4c. This demonstrates that water does not react with the Al in I-Al/PVDF. This is due to the fact that Al is contained in the gel blocks produced by disordered PVDF, as seen in Figure 1a,b. Then, after a solvent exchange and freeze-drying, Al was encapsulated by PVDF. This explains why I-Al/PVDF exhibits strong anti-aging properties underwater.

### 2.4. Thermal Analysis

**Figure 5:**
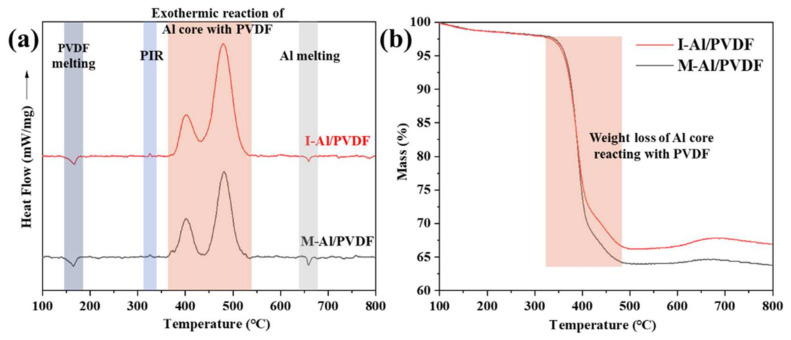
(**a**) DSC traces and (**b**) TGA traces of I-Al/PVDF and M-Al/PVDF.

As shown in Figure 5, the reaction pathways of I-Al/PVDF and M-Al/PVDF were investigated by DSC/TGA experiments. For each sample, three exothermic peaks (~325 °C, ~400 °C, ~480 °C) and two endothermic peaks (~167 °C, ~660 °C) were observed. The two endothermic peaks correspond to the melting peaks of PVDF and Al, respectively. The pre-ignition reaction (PIR) of the Al_2_O_3_ shell with HF produced by PVDF breakdown is represented by the exothermic peak at 325 °C [28,29]. PIR is found in a wide range of energetic n-Al/fluoropolymer combinations, including Al/PTFE, Al/PFPE, and Al/PVDF. Al nanoparticles profit from PIR since the Al_2_O_3_ shell layer is pre-fluorinated, which facilitates the ability to dissolve the barrier between the new Al core and PVDF and eventually encourages thermal reactions [30,31]. The two larger exothermic peaks at ~395 °C and ~480 °C correspond to the reaction between the decomposed PVDF and the Al particle core [32].

As shown in Figure 5b, the TGA results illustrate that the percentage change in sample mass is smaller for I-Al/PVDF compared to M-Al/PVDF. This indicates that the reaction of I-Al/PVDF is more complete. Because if PVDF does not react with Al, it will decompose and form gaseous products (such as HF, CF_x_, and C_x_H_y_F_z_), which escape and cause mass loss [32]. This is consistent with the larger exothermic peak and smaller Al melting peak of I-Al/PVDF in DSC. The reason for this phenomenon may be due to the less agglomeration of n-Al in I-Al/PVDF compared with M-Al/PVDF, and the more adequate interfacial contact between n-Al and PVDF, as shown in Figure 1. This leads to a more complete reaction between PVDF and n-Al in I-Al/PVDF under temperature programmed.

### 2.5. Underwater Ignition Tests

**Figure 6:**
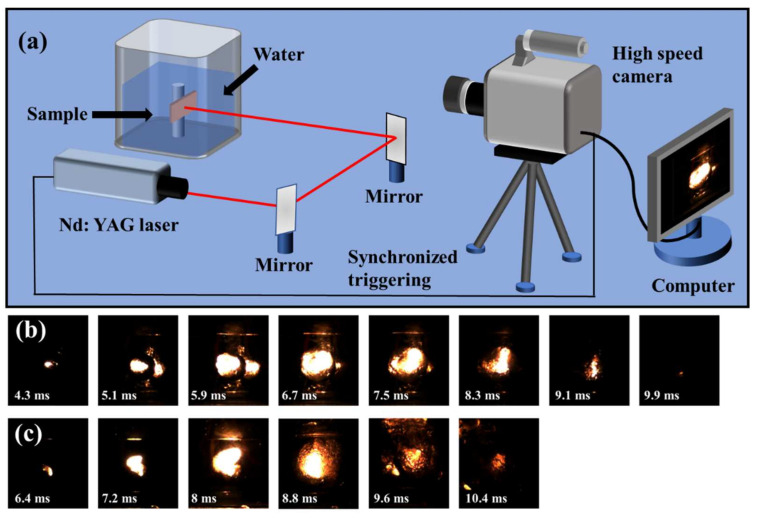
(**a**) The schematic diagram of the submerged ignition process; The snapshot for the submerged laser ignition process of the (**b**) I-Al/PVDF and the (**c**) M-Al/PVDF.

**Figure 7:**
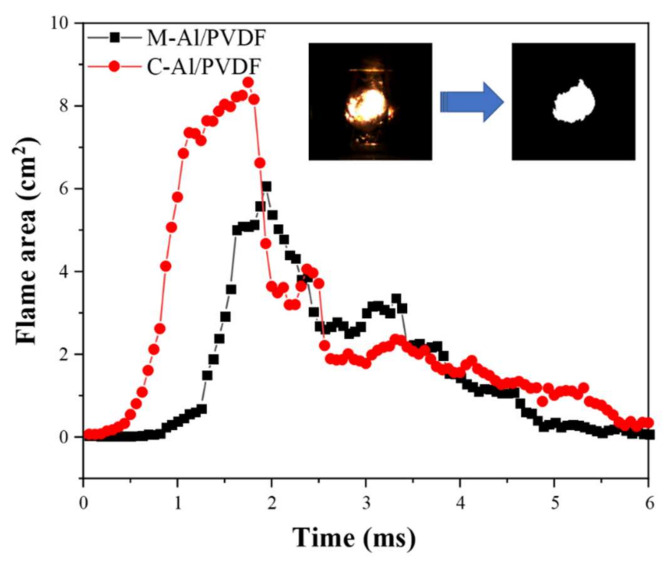
The curve of flame area versus time for samples M-Al/PVDF and C-Al/PVDF.

Al-fluoropolymer systems have been studied for their reactivity [33,34,35,36], but their capability to ignite underwater has not received as much attention. In this work, the underwater ignition performance of I-Al/PVDF and M-Al/PVDF was studied using a laser ignition source and a high-speed camera. The schematic diagram of the experimental process is shown in Figure 6a. The mass of the I-Al/PVDF and M-Al/PVDF membranes used for testing is 3.2 mg, and the densities are 0.53 g·cm^−3^ and 0.84 g·cm^−3^, respectively. Compared with M-Al/PVDF, I-Al/PVDF has a larger flame area and a shorter time to reach the maximum flame area, as shown in Figure 6b,c and Figure 7. This may be because the porous structure on the surface of the energetic film is favorable for the absorption of laser light [30]. Meanwhile, I-Al/PVDF exhibits longer energy release time. This may be due to the good dispersibility of fuel and oxidant in I-Al/PVDF. Additionally, Al is well coated by PVDF, which can effectively resist aging, as mentioned above. As a result, the samples prepared by the cryogel process can release energy relatively well in the aqueous environment.

## 3. Materials and Methods

### 3.1. Materials

Al nanopowder (with an average particle size of 80 nm) was purchased from Naiou Nano Technology Co., Ltd (Shanghai, China). The results of TG showed that the content of active aluminum was 73%, as shown in Figure S1. PVDF (Mw of 250,000–500,000) was purchased from China National Pharmaceutical Group. Anhydrous ethanol and N, N-dimethylformamide (DMF, 99.8%) were purchased from Sinopharm Chemical Reagent Co., Ltd. (Shanghai, China), and were used as received without further purification.

### 3.2. Preparation of n-Al/PVDF Energetic Thin Films

**Figure 8:**
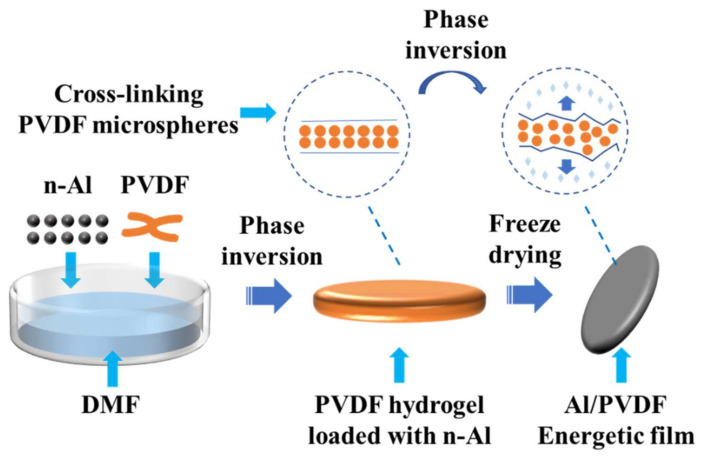
Schematic diagram of the preparation process.

Figure 8 shows a schematic diagram of the n-Al/PVDF energetic film prepared by the cryogel-templated process. Aluminum nanoparticles (Al NPs) were put into PVDF/DMF solution (10% wt) and, after sonication, vigorously magnetically stirred for 24 h to obtain a homogeneous precursor solution. The precursor solution was gradually transformed into a gel by adsorbing ethanol gas. The gel was soaked in ethanol and then in water for solvent exchange to remove DMF. Finally, the gel was dried at −60 °C under vacuum (<10 Pa) to obtain n-Al/PVDF energetic films. The energetic films prepared by the cryogel-templated process were named I-Al/PVDF. The pure PVDF film prepared by the cryogel-templated process was named I-PVDF. The Al/PVDF films prepared by the mechanical mixing-casting method were named M-Al/PVDF. The mechanical mixing–casting method is to directly cast the aforementioned Al/PVDF precursor solution to form a film. All Al/PVDF composites were set to a stoichiometric ratio (ϕ = 1, 30 wt% Al and 70 wt% PVDF).

### 3.3. Characterizations Methods

The X-ray diffraction (XRD) patterns of the obtained materials were acquired using a Bruker D8 Advance (Bruker, Billerica, MA, USA) equipped with monochromatized Cu Kα radiation (λ = 0.15406 nm). The morphologies and structures of all samples were characterized using a FEI field-emission scanning electron microscope which equipped with an energy dispersive X-ray spectrometer (EDS) by Quanta FEG 250 (Field Electron and Ion Company, Hillsboro, OR, USA). Differential scanning calorimetry (DSC, TGA/DSC 3+, METTLER TOLEDO, Greifensee, Switzerland) was used for thermal analysis with a heating rate of 20 °C·min^−1^ and an Ar flow of 30.0 mL·min^−1^. The water contact angle and water rolling angle were tested by a goniometer (Powereach JC2000D, Shanghai, China). Measurements were performed using and 5 μL droplets of deionized water. Data were obtained by measuring the mean of at least three distinct points in each sample.

### 3.4. Combustion Characterization

All ignition experiments were recorded with a high-speed camera (Photron MiniUX50, Tokyo, Japan) at 10,000 fps. A Q-switched Nd:YAG laser pulse (1064 nm, 6.5 ns, 8.7 mJ·cm^−2^, Maxphotonics, China) was used in the laser ignition process. The calculation of the flame area is achieved by processing high-speed photographic images in MATLAB. In general, the processing process of MATLAB is divided into three steps: (1) remove the dark background; (2) find the number of pixels in the largest bright connected region in the image; and (3) calculate the pixel area according to the scale.

## 4. Conclusions

To summarize, this paper reports the application of cryogel-template fabrication in the preparation of Al/PVDF energetic thin films. Through a facile and safe process, Al/PVDF energetic films with superhydrophobic surfaces were prepared. The film’s aluminum nanoparticles are uniformly encased in PVDF, giving it excellent anti-aging capabilities. The Al/PVDF energetic film has a strong heat release ability, according to thermal analysis. Laser-induced underwater ignition experiments show that the superhydrophobic modification can effectively induce the ignition of energetic films underwater. These results suggest that the cryogel-template fabrication provides a feasible route for the application of energetic materials at the underwater microscale.

## Data Availability

Not applicable.

## Supplementary Materials

The following supporting information can be downloaded at: https://www.mdpi.com/article/10.3390/molecules27206911/s1, Figure S1: TGA curve of Al-NPs in air.; Figure S2: Digital photographs of Al NPs, M-Al/PVDF and I-Al/PVDF films before and after aging experiment.
