# Supplementary material for: Cryogel-Templated Fabrication of n-Al/PVDF Superhydrophobic Energetic Films with Exceptional Underwater Ignition Performance
Source: Molecules. 2022 Oct 14;27(20):6911. doi: 10.3390/molecules27206911 (PMC9611366; doi:10.3390/molecules27206911)
Supplement: Supplementary file 1 [file molecules-27-06911-s001.zip › molecules-1952649-supplementary.pdf]

Cryogel-templated fabrication of Al/PVDF superhydrophobic energetic films with exceptional underwater ignition performance

Jingwei Li 1,2,3, Xuwen Liu 1,2,3*, Quanmin Xie 1,2, Yongsheng Jia 1,2, Jinshan Sun 1,2, Yingkang Yao 1,2

1 State Key Laboratory of Precision Blasting, Jianghan University, Wuhan 430056, China

2 Hubei Key Laboratory of Blasting Engineering of Jianghan University, Wuhan, 430056

3 School of Chemistry and Chemical Engineering, Nanjing University of Science and Technology, Nanjing 210094, China

* Correspondence: lxw@njust.edu.cn; Tel.: +86 18655488806

Experimental details.

1. Calculation of the active Al content in Al NPs

The oxidation of active aluminum is the cause of the mass gain in the TGA, which is represented by the TGA curve in Fig.S1. The following equation can be used to get the active aluminum content (c):

$$c(\%) = \frac{108}{96}\Delta m(\%)$$

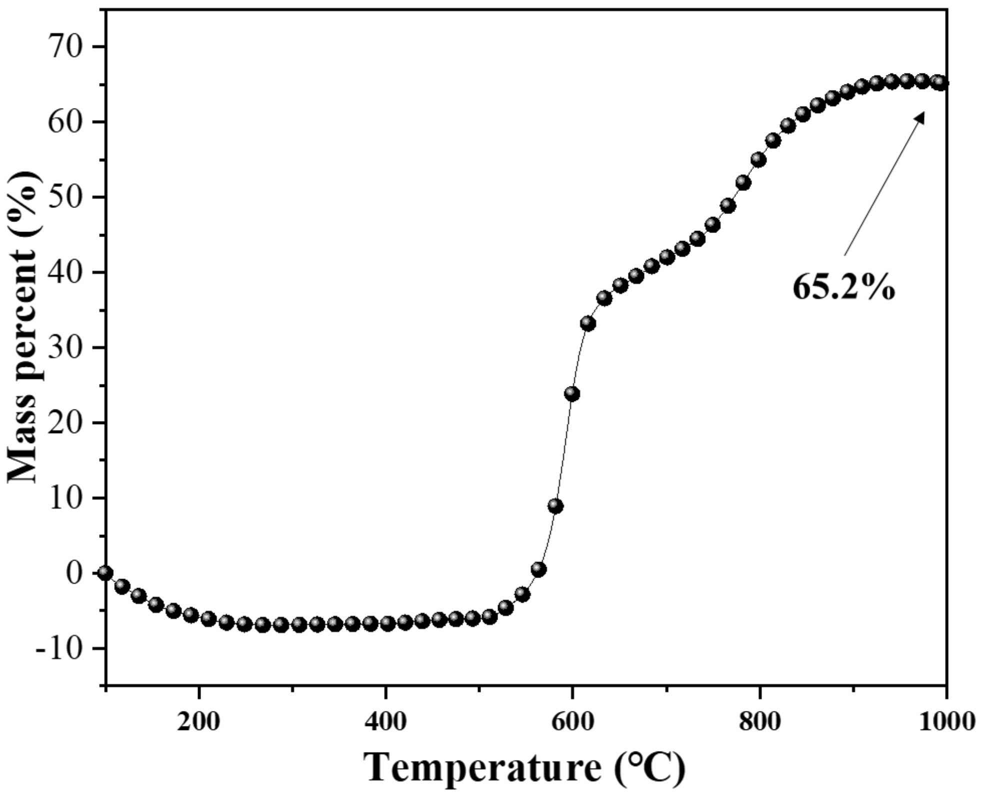

Figure S1. TGA curve of Al-NPs in air.

2. Digital photographs of aging experiment

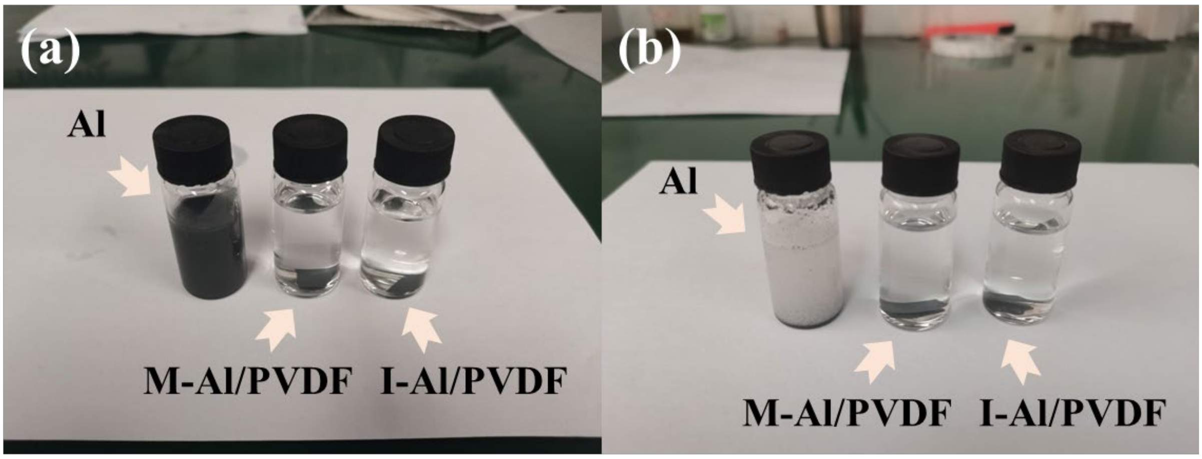

Figure S2. Digital photographs of Al NPs, M-Al/PVDF and I-Al/PVDF films (a) before and (b) after aging experiment.

As shown in Fig. S2(a), the Al NPs, M-Al/PVDF and I-Al/PVDF all appear light black before aging. But after aging, as shown in Fig. S2(b), the light black color of the Al-NPs suspension changed to off-white.

This is because the Al reacts with the water in the water to form a white flocculent precipitate of aluminum hydroxide with hydrogen:

$$Al + H_2O \rightarrow Al(OH)_3 + H_2$$

However, the light black of the I-Al/PVDF and M-Al/PVDF films has no color change, which directly shows its remarkable anti-aging performance.
